# Thymoquinone Induces Mitochondria-Mediated Apoptosis in Acute Lymphoblastic Leukaemia *in Vitro*

**DOI:** 10.3390/molecules180911219

**Published:** 2013-09-12

**Authors:** Landa Zeenelabdin Ali Salim, Syam Mohan, Rozana Othman, Siddig Ibrahim Abdelwahab, Behnam Kamalidehghan, Bassem Y. Sheikh, Mohamed Yousif Ibrahim

**Affiliations:** 1 Department of Pharmacy, Faculty of Medicine, University of Malaya, KualaLumpur 50603, Malaysia; 2 Medical Research Centre, Jazan University, P.O. Box 114 Jazan, Jazan 45142, Saudi Arabia; 3 College of Medicine, Taibah University, Al Madinah Al Monawarah 30001, Saudi Arabia

**Keywords:** anticancer, apoptosis, mitochondria, thymoquinone, CEMss

## Abstract

There has been a growing interest in naturally occurring compounds from traditional medicine with anti-cancer potential. *Nigella sativa* (black seed) is one of the most widely studied plants. This annual herb grows in countries bordering the Mediterranean Sea and India. Thymoquinone (TQ) is an active ingredient isolated from *Nigella sativa*. The anti-cancer effect of TQ, via the induction of apoptosis resulting from mitochondrial dysfunction, was assessed in an acute lymphocyte leukemic cell line (CEMss) with an IC_50_ of 1.5 µg/mL. A significant increase in chromatin condensation in the cell nucleus was observed using fluorescence analysis. The apoptosis was then confirmed by Annexin V and an increased number of cellular DNA breaks in treated cells were observed as a DNA ladder. Treatment of CEMss cells with TQ encouraged apoptosis with cell death-transducing signals by a down-regulation of Bcl-2 and up-regulation of Bax. Moreover, the significant generation of cellular ROS, HSP70 and activation of caspases 3 and 8 were also observed in the treated cells. The mitochondrial apoptosis was clearly associated with the S phase cell cycle arrest. In conclusion, the results from the current study indicated that TQ could be a promising agent for the treatment of leukemia.

## 1. Introduction

*Nigella sativa*, also known as black cumin (black seed), is an annual herbaceous plant belonging to the Ranunculacea family which grows in countries bordering the Mediterranean Sea and India [1]. It has been used as traditional herbal medicine for more than 2,000 years [2]. Apart from this complimentary medicinal use, it has been used in Asia, Middle East and Africa as a medicinal food to support health and fight many diseases [3]. *N. sativa* is one of the most extensively studied plants [4]. It used as a food additive and flavour worldwide [2,5]. They are also used as a natural remedy for asthma, hypertension, diabetes, inflammation, cough, bronchitis, headache, eczema, fever, dizziness and influenza [4,6]. The seeds are known to be carminative, stimulant, diuretic, emenagouge and galactogogue, and have been used in treating fever [7]. The biological activity of *N. sativa* seeds is attributed to their essential oil components [8]. The main compounds contained are thymoquinone (30%–48%), *p*-cymene (7%–15%), carvacrol (6%–12%), 4-terpineol (2%–7%), *t*-anethole (1%–4%) and the sesquiterpene longifolene (1%–8%) [9].

**Figure 1:**
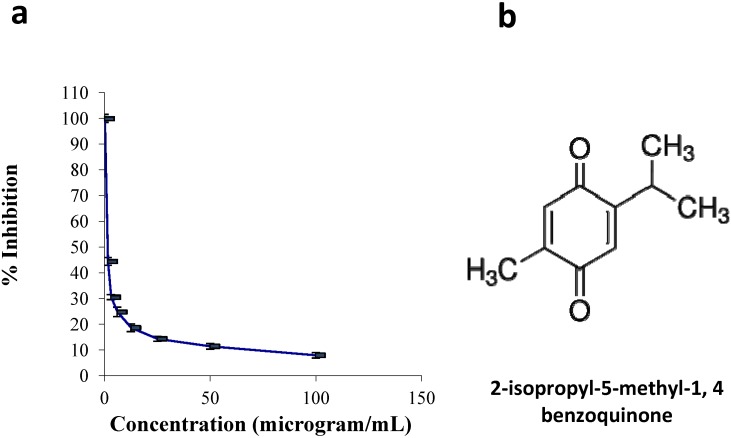
Effects of Thymoquinone on cell viability in CEMss. (**a**) The cell viability of cells after 24 h of treatment. Each point is the mean ± S.D. of three independent experiments. (**b**) The chemical structure of thymoquinone.

In Malaysia, *N. sativa* is popularly known as “el Habbatus el Sauda”. Currently, the black seed oil has been widely commercialized and due to its amazing healing power, numerous research projects are being carried out from time to time to improve its quality [10,11]. Thymoquinone (TQ, 2-isopropyl-5-methyl-1,4-benzoquinone, Figure 1b) is the major component and the most bioactive constituent of the volatile oil of this seed, which has been shown to possess anti-inflammatory, antioxidant and anti-carcinoma effects [12]. In recent years, TQ and its effects on different cancer cell lines have been widely studied; these effects include inhibition of cancer cell viability. In almost all pancreatic cancer cell lines tested, the inhibition was up to 70% [13]. Anti-proliferative and pro-apoptotic activities of TQ in both NSCLC and SCLC cell line [14]. Breast cancer cell lines [15] and liver cell lines [16] have also been studied, including in some animal models [17].

The problem of cancer burden in Malaysia is growing, as in other countries around the World, since it is one of the major health problems, which leads to death [18]. Leukaemia is one of the most common childhood cancers worldwide and also in Malaysia [19]. Childhood leukaemia represents 4.7% of childhood cancers in the Malaysian population [20]. This is treated by multidisciplinary efforts including chemotherapy. One of the main problems of the present chemotherapy in treating tumour patients is the toxicity of the drugs used. Most of the existing anticancer drugs, unfortunately, also attack normal proliferating cells [21]. Therefore, being generally considered as safe, consumption of natural compounds is currently a major interest in health care. In the current study, we evaluated the potential of TQ on T acute lymphoblastic leukaemia using CEMss cells as an *in vitro* model.

## 2. Results and Discussion

### 2.1. Results

#### 2.1.1. Cell Culture and Viability Assay

The effects of TQ on the viability of CEMss cells were measured using the MTT assay. Cellular proliferation following 24 h of exposure to TQ showed significant inhibition in TQ-treated cells compared to non-treated cells (controls). As shown in Figure 1, the IC_50_ of TQ was 1.5 ± 0.04 μg/mL following 24 h of treatment. The proliferation of TQ-treated cells decreased as the TQ concentration increased.

#### 2.1.2. Quantification of Apoptosis Using Propidium Iodide and Acridine Orange Double-Staining

**Figure 2:**
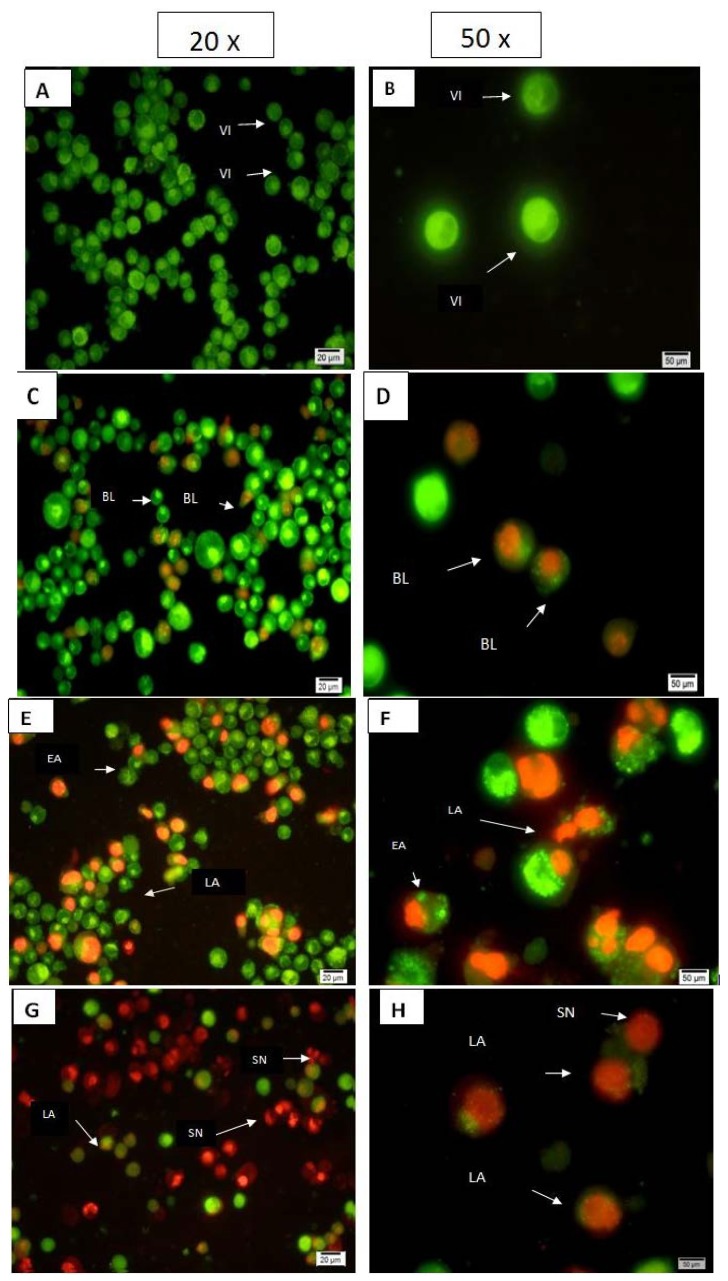
Fluorescent micrographs of acridine orange and propidium iodide double- stained CEMss cells. Cells were treated at the IC_50_ of thymoquinone in a time-dependent manner. Cells were cultured in RPMI 1640 media maintained at 37 °C and 5% CO_2_. (**A**,**B**) Untreated cells after 72 h showed normal structure without prominent apoptosis and necrosis. (**C**,**D**) Early apoptosis features were seen after 24 h representing intercalated acridine orange (bright green) amongst the fragmented DNA, (**E**, **F**) Blebbing and orange color representing the hallmark of late apoptosis were noticed in 48 h treatment, (**G**,**H**) bright red colored secondary necrosis were visible after 72 h. VI: viable cells; BL: blebbing of the cell membrane; LA: late apoptosis; SN: secondary necrosis. Images are representative of one of three similar experiments.

**Figure 3:**
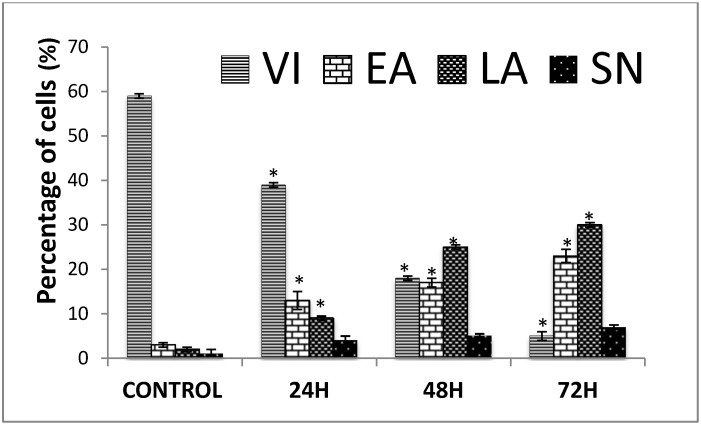
Percentages of viable, early apoptotic, late apoptosis and secondary necrotic cells after thymoquinone treatment. CEMss cell death via apoptosis increased significantly (* *p* < 0.05) in a time-dependent manner. However, no significant (*p* > 0.05) difference was observed in the cell count of necrosis. VI: viable Cell, EA: Early apoptosis, LA: late apoptosis, SN: secondary necrosis.

Apoptotic, necrotic and viable CEMss cells were scored under the fluorescence microscope. These also included the control cells (untreated); 200 cells were randomly and differentially counted. The study revealed that TQ triggered morphological features that relate to apoptosis in a time-dependent manner (Figure 2). Early apoptosis was obvious by intercalated AO within the fragmented DNA. In several such cases, the fluorescent bright-green colour could only be seen in treated CEMss cells. In contrast, untreated cells were observed to have a green intact nuclear structure. At 24 h treatment with TQ, blebbing and nuclear margination were noticed (moderate apoptosis). In addition, late stages of apoptosis (*i.e.*, presence of orange colour due to the binding of AO to denatured DNA) were observed after a 48 and 72 h treatment with TQ (Figure 2). Differential scoring of treated CEMss cells (200 cell population) as exhibited in Figure 3 showed that there was a statistically significant (*p* < 0.05) difference in apoptotic positive cells, which indicated clearly that TQ has a time-dependent apoptogenic effect. On the other hand, there was no statistically significant (*p* > 0.05) difference in necrotic counts at different times during treatment (24, 48, and 72 h), as displayed in Figure 2.

#### 2.1.3. Annexin V

**Figure 4:**
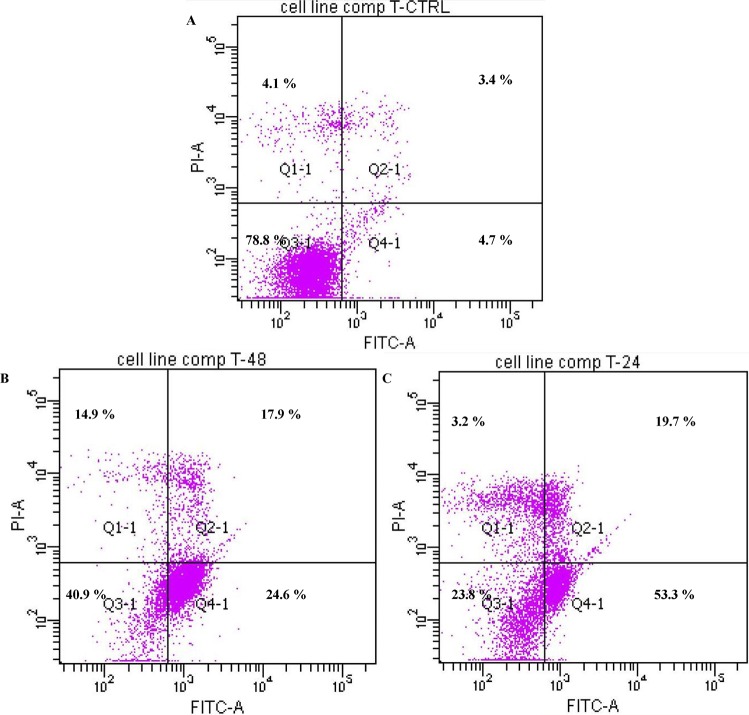
The effect of thymoquinone on early apoptosis of CEMss cells. CEMss cells were exposed to1.5 µg/mL and incubated at 37°C in a CO_2_ incubator. After staining with FITC-conjugated Annexin V and PI, cells were analyzed by flow cytometry. Control cells received no drug treatments. The early apoptotic events (Annexin+/PI-) are shown in lower right quadrant (Q4-1) of each panel. Quadrant (Q2-1) represents Annexin+/PI+ late stage of apoptosis/dead cells. (A) The CEMss control (n = 2). (A–C) The effects of 0, 24 and 48 h exposure (respectively) of CEMss cells to Thymoquinone.

The induced apoptotic effect of TQ was further confirmed by the determination of the percentage of apoptotic cells using flow cytometric analysis with the AV/PI double staining. The AV+/PI_ staining represents the early apoptotic cells due to the strong affinity of AV-FITC with phosphatidylserine, which transports from the inner leaflet of the plasma membrane to the outer surface of the membrane during early apoptosis. On the other hand, AV_/PI+ staining represents the necrotic cells, since PI, which could not cross through an intact cell membrane, penetrates the compromised membrane of dead cells or late apoptotic cells and binds to nucleic acid. Meanwhile, viable cells can be marked by AV_/PI_, and AV+/PI+ staining is indicative of late apoptotic cells. The representative dot plots of the flow cytometric analysis of apoptosis showed that based on a comparison between untreated cells (control) and treated cells (24 h and 48 h), the percentages in early apoptosis and late apoptosis respectively increased (Figure 4). In addition to that, TQ treatment clearly produced a slight decrease in viable cells at 24 and 48 h. The results further suggest that the antiproliferative effect of TQ against CEMss cells is caused by inducing cell apoptosis.

#### 2.1.4. DNA Fragmentation Assay

**Figure 5:**
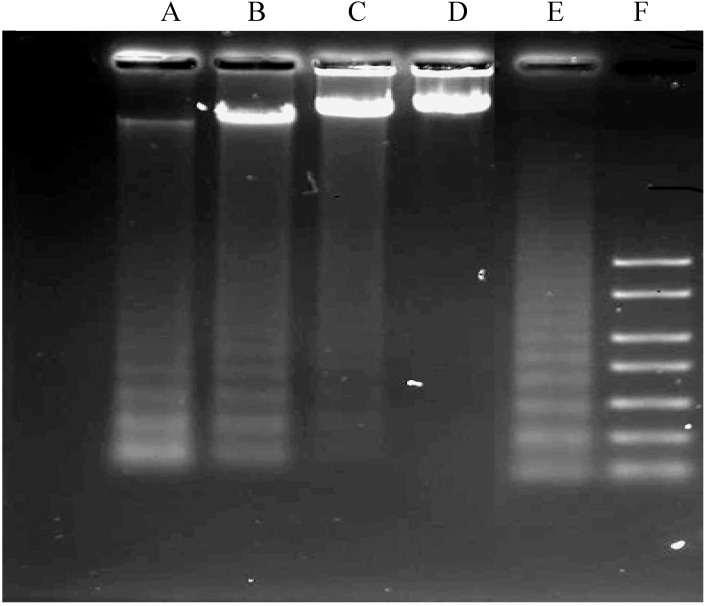
Effects of Thymoquinone on DNA fragmentation in CEMss cells. (Lanes A–D were treated with 1.5 µg/mL. Lane E: positive control and lane F: marker). Figure shows data representative of three independent experiments.

The DNA ladder pattern of internucleosomal fragmentation (Figure 5), another characteristic of apoptosis, was distinctly seen in TQ-treated cells. Formation of the DNA ladder pattern also increased in a time-dependent manner. Positive control in the figure shows the ladder of the DNA whilst the negative control shows the untreated cells which did not undergo apoptosis. The ladder visibly increased in a time-dependent manner.

#### 2.1.5. Caspases 3, 8 and 9 Analyses

**Figure 6:**
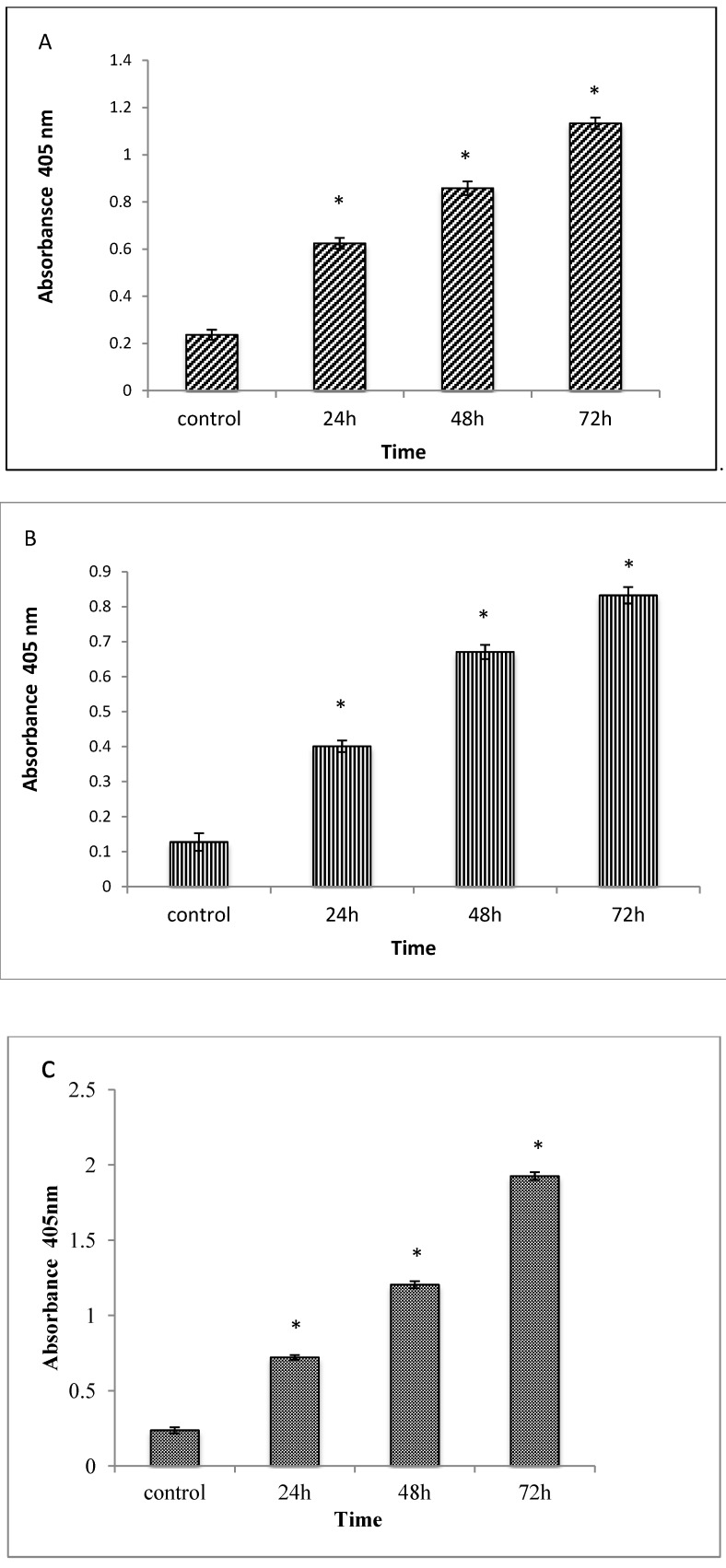
Relative expression of Caspase-3 (A), -8 (B) and -9 (C) in the CEMss cells treated with different concentrations of Thymoquinone. Triplicates of each treatment group were used ineach independent experiment. The statistical significance is expressed as *, *p* < 0.05. A: Caspase 3, B: Caspase 8, C: Caspase 9.

The apoptotic effect of TQ was also examined by measuring the activities of caspases 3, 8 and 9 enzymes. The enzyme activities were determined in relation to the different concentrations of protein content (50–250 μg/sample) for cells treated with IC₅₀ of TQ. The results obtained showed an increase of the enzyme activities in a time-dependent manner [22], as shown in Figure 6.

#### 2.1.6. Cell Cycle Analysis

**Figure 7:**
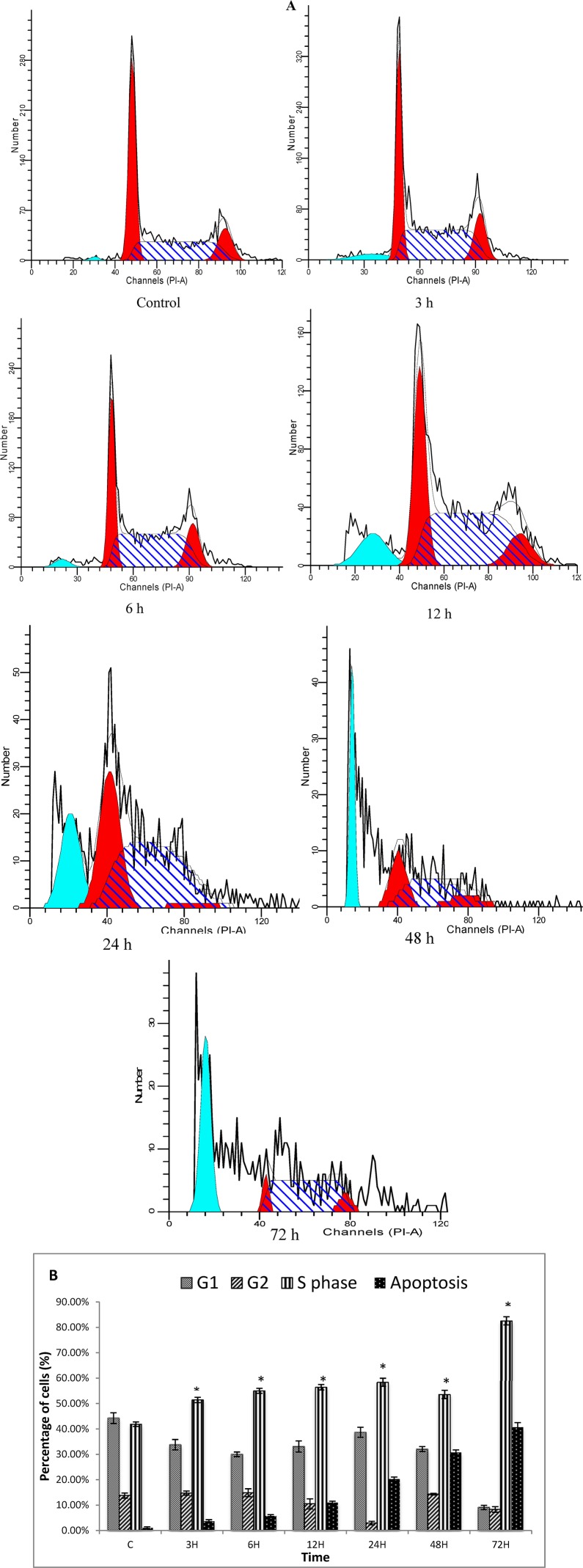
Histograms for cell cycle from analysis of CEMss cells treated with thymoquinone (1.5 µg/mL) (**A**). Results are representative of one of three independent experiments. Induction of S phase arrest in the cell cycle progression of CEMss cells thymoquinone (**B**). “*” Indicates a significant difference *p* < 0.05.

Both treated and untreated cells were analyzed in terms of cell cycle distribution by means of flow cytometry. The cell cycle analysis confirmed that TQ induced a depletion of cells in the G1 phase. However, apoptosis increased significantly (Figure 7A) at 3, 6, 12, 24, 48 and 72 h of treatment, which indicated that these cells had undergone apoptosis (Figure 7). These results suggest that TQ induces S phase cell cycle arrest followed by apoptosis in CEMss cells [23].

#### 2.1.7. Western Blots

**Figure 8:**
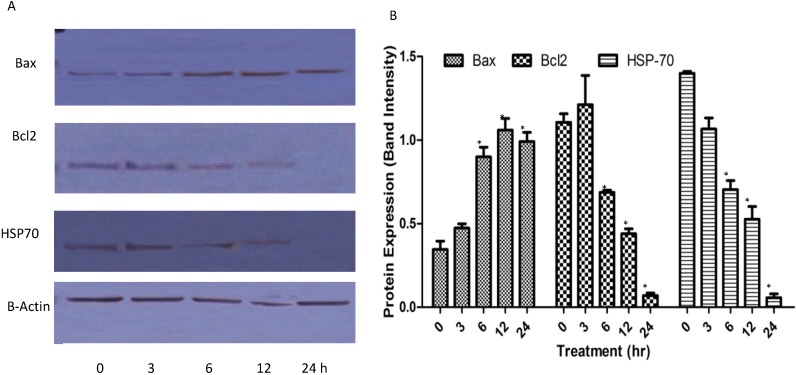
Effect of TQ on the levels of apoptosis regulatory proteins at 3, 6, 12 and 24 h with β- actin as a loading control. ‘*’ indicates statistically significant at *p* < 0.05.

Bcl-2 and Bax are members of the Bcl-2 family that play key roles in the regulation of apoptosis; these proteins are believed to be membrane-bound and their ability to undergo both homodimerization and heterodimerization has been proposed to regulate apoptosis [24]. Although the caspase proteolytic cascade is a central point in apoptotic response, its activity is tightly regulated by a variety of factors. Among these, Bcl-2 family proteins, including anti-apoptotic members (such as Bcl-2) and pro-apoptotic members (such as Bax and Hsp70), play a pivotal role. To further analyze the possible mechanism underlying the TQ-induced apoptosis, we tested the expression of Bcl-2, Bax and Hsp70 in CEMss cells after the TQ treatment. After being normalized to β-actin, the expression of Bcl-2 and Hsp70 decreased significantly while Bax protein level increased remarkably in a dose-dependent manner (Figure 8). The ratio of Bax: Bcl-2 Hsp70 also dramatically increased in a time-dependent manner [25].

#### 2.1.8. Reactive Oxygen Species (ROS)

**Figure 9:**
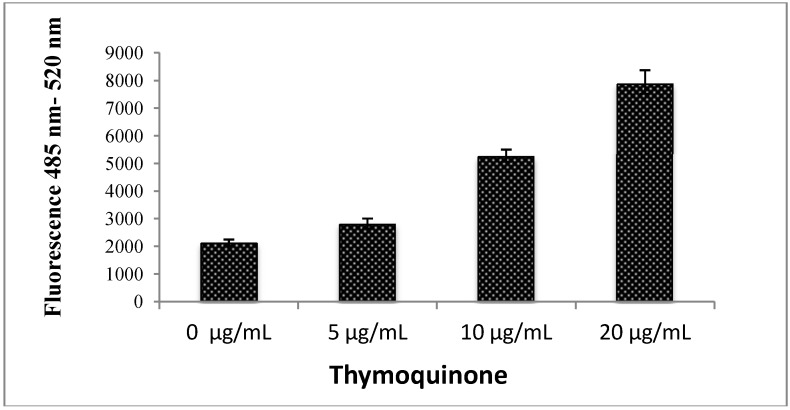
Effect of thymoquinone treatment on ROS generation. ROS concentration was determined by treating CEMss cells with 0, 5, 10 and 20 μg/mL TQ, the cells were loaded with DCFH-DA, and the fluorescence was measured by a fluorescence micro plate reader.

Reactive oxygen species (ROS) are a variety of molecules and free radicals derived from molecular oxygen which are constantly generated and eliminated in the biological system and have important roles in cell signalling and homeostasis. Excessive amounts of ROS can cause oxidative damage to lipids, proteins and DNA, leading to tumour genesis or cell death. Although the use of antioxidants in humans for cancer prevention remains controversial, increasing evidence have supported that the increase of ROS generation contributes to the treatment of cancer cells. As shown in Figure 9, we found that TQ exerted a facilitator role in the production of ROS in a concentration-dependent manner, which increased the ROS level on CEMss cell lines [26].

### 2.2. Discussion

Apoptosis is one form of physiological or active cell death, or a key pathway for regulating homeostasis and morphogenesis of mammalian cells and is connected with several diseases, especially cancer [27]. It describes the orchestrated collapse of a cell characterized by membrane blabbing, cell shrinkage, condensation of chromatin, and fragmentation of DNA followed by a rapid engulfment of the corpse by neighbouring cells [28]. Apoptosis is becoming widely acknowledged as being an innate tissue protection against carcinogens by inhibiting survival and controlling the growth of precancerous cell populations and tumours at different stages of carcinogenesis. Reflecting on this knowledge, the mechanism of action for many currently used anticancer agents have been specifically targeted to regulate the apoptotic pathway, further stressing the role of programmed cell death in maintaining normal homeostasis. A major concern of cancer chemotherapy is the side effects caused by the non-specific targeting of both normal and cancerous cells by therapeutic drugs. Much emphasis has been placed on discovering new compounds that target tumour cells more efficiently and selectively with minimal toxic effects on normal cells [29]. Therefore, several researchers nowadays have performed anti-cancer studies on herbal extracts as well as natural compounds based on their biochemical properties of apoptosis [30,31].

Thymoquinone is the major bioactive constituent present in black seed oil (*Nigella sativa*) and has been tested for its efficacy against cancer [16,32,33]. Many researchers have studied TQ’s mechanism of action and its aptitude to induce apoptosis and inhibit tumour growth [34]. To date, the chemotherapeutic potential of TQ in the clinic has not been tested [35]. However, numerous studies have shown its promising anti-cancer effects in animal models [36]. In the present research we have shown that the cyctotoxic activities of TQ towards CEMss cells are selective (Figure 1). Since the cytotoxicity was found to be selective, we extended the study to determine the mechanism of cytotoxicity. Acridine orange (AO) and propidium iodide (PI) are among the most used fluorescent dyes to analyse cell culture viability and morphological charecteretics. We have observed various stages of apoptosis, starting from chromatin condensation till apoptotic body formation with TQ treatment. Even though the morphological features were clearly observed, in an attempt to quantify the cells of apoptotic population the annexin V assay was performed. Annexin V was shown to interact strongly and specifically with PS and could be used to detect apoptosis by targeting the loss of plasma membrane asymmetry [37]. The present study exhibits that treatment with TQ is able to induce cell death via apoptosis in human CEMss cells. The results showed that there was a significant time-dependent increase in the early stage of apoptosis, as shown clearly in Figure 4. In addition to annexin V analysis, the late stage of apoptosis has been confirmed by using a DNA laddering assay, which will identify the cleavage of chromosomal DNA into oligonucleosomal-sized fragments is an integral part of apoptosis [38]. Moreover this assay was usded before many time to detect the apoptosis in leukemia cell lines [39,40].

Apoptosis is orchestrated by a family of cysteine proteases known as caspases. The main effectors of apoptosis encompass proteases from the caspase family, which reside as latent precursors in most nucleated animal cells [41]. Fourteen mammalian caspases have been identified, three of which (caspase-3, -6, and -7) are thought to coordinate the execution phase of apoptosis by cleaving multiple structural and repair proteins [42]. Pathways to caspase-3 activation have been identified, which are either dependent on or independent of mitochondrial cytochrome c release and caspase-9 function. Caspase-3 is required for typical hallmarks of apoptosis, and is indispensable for apoptotic chromatin condensation and DNA fragmentation in all cell types examined. Thus, caspase-3 is essential for certain processes associated with the dismantling of the cell and the formation of apoptotic bodies, but it may also function before or at the stage when commitment to loss of cell viability is made [43]. Caspase-8 is a family member of executioner caspases associated with tumour necrosis factor (TNF) family death receptors-mediated apoptotic signalling cascade [44]. In this study, the results of caspases 3, 8 and 9 activities significantly showed that TQ induces apoptosis in CEMss in a time-dependent manner which is caspase dependent.

Phytochemicals that function as cell-cycle modulators are gaining widespread attention due to the evidence of concomitant involvement of apoptosis and cell cycle inhibition. The cell cycle has four sequential phases. Arguably, the most important phases are the S phase, when DNA replication occurs, and the M phase, when the cell divides into two daughter cells. During the past two decades, cancer genetics have shown that hyperactivating mutations in growth signalling networks, coupled with loss of function of tumour suppressor proteins, drive oncogenic proliferation. The cell cycle machinery, which acts as an integration point for information transduced through upstream signalling networks, represents an alternative target for diagnostic and therapeutic interventions. Analysis of the DNA replication initiation machinery and mitotic engine proteins in human tissues is now leading to the identification of novel biomarkers for cancer detection and prognostication, and is providing target validation for cell cycle-directed therapies [45]. Thus flow cytometry analysis was performed to analyze the various cell cycle check points at TQ treatment. Our results indicate that cell cycle distribution is altered in CEMss cells after TQ treatment. This growth inhibition was mediated through blocking progression of the leukemia cells through the S phase of the cell cycle, as previously described [46].

Reactive oxygen species (ROS) are constantly generated and eliminated in the biological system, and play important roles in a variety of normal biochemical functions and abnormal pathological processes. Consequently, humans have evolved antioxidant defence systems that limit their production. Generation of oxidative stress in response to various external stimuli has been implicated in the activation of transcription factors and with the triggering of apoptosis [47]. Growing evidence suggests that cancer cells exhibit increased intrinsic ROS stress, due in part to oncogenic stimulation, increased metabolic activity and mitochondrial malfunction. Previously TQ-induced apoptosis of chondrocytes has been reported to be involved in the generation of ROS [48]. In agreement with this we found that TQ is a potent inducer of apoptosis in CEMss cells via release of ROS. The generation of ROS is cloely associated with mitochondrial proteins such as Bcl-2 and Bax. These proteins are believed to be membrane-bound and their ability to undergo both homodimerization and heterodimerization has been proposed to regulate apoptosis [49]. The Bcl-2 family proteins are key regulators of apoptosis, which include both anti- and pro-apoptotic proteins and a slight change in the dynamic balance of these proteins, may result in either inhibition or promotion of cell death [50]. Meanwhile, there are many prtoiens inside the cells which will prevent the ROS mediated cell dammage. Amongst them, HSP70 is widely studies for its protection fuction. It is a chaperone that accumulates in the cells after many different stresses promoting cell survival in response to the adverse conditions. In contrast to normal cells, most cancer cells richly express HSP70 at the basal level to resist various insults at different stages of tumourigenesis and during anti-cancer treatment [51]. We have found that TQ caused a significant down-regulation of Bcl2 and HSP70 and an up-regulation of BAX in a time-dependent manner. 

## 3. Experimental

### 3.1. Chemicals and Reagents

Thymoquinone (>99% pure) was purchased from Sigma (St. Louis, MO, USA). CEMss cell lines used in this study were obtained from the NIH AIDS Research and Reference Reagent Program, Division of AIDS, NIAID, NIH: Bethesda, MD, USA. RPMI 1460, fetal bovine serum (FBS) and penicillin-streptomycin were obtained from Bioscience Ltd. (Wokingham, UK). Phosphate buffered saline (PBS), ethanol (95%), DNA laddering kit, 3-(4,5-dimethylthiazol-2yl)-2,5-diphenyltetrazolium bromide (MTT) and DCFH-DA (2'-7'-dichlorodihydrofluorescein diacetate were from Santa Cruz Biotechnology (Santa Cruz, CA USA), propidium iodide and acridine orange was purcahed from Nacalai Tesque (Japan), Annexin V kit was from ClonTech (Palo Alto, CA, USA).

### 3.2. Cell Culture and Viability Assay

Cell cultures were maintained in a humidified atmosphere with 5% CO_2_ at 37 °C. To study proliferation and determine the survival of cancer cells after treatment, the MTT assay was used. Briefly, the cells were plated on a 96-well plate at 2 × 105 cell/mL in 100 μL culture medium. They were plated in triplicate. Different concentrations of TQ (50, 25, 12.5, 6, 3 and 1.5 µg/mL) were prepared by serial dilution. All serial dilutions were transferred to the cells in the 96-well plate; the plate included untreated cells as control and was incubated for 24 h. After incubation, the viability of the cells was assessed using 3-[4,5-dimethylthiazol-2-yl]-2,5-diphenyltetrazolium bromide (MTT, 5 mg/mL); 20 μL were added to the cells in a dark place, and the cells were then covered with aluminium foil and incubated for 4 h. After incubation, all of the media were removed and 100 μL of DMSO were added to the cells in order to solubilise the formazan crystals. Subsequently, the absorbance was read at a wavelength of 570 nm using a micro plate reader. The potency of cell growth inhibition for the test agent was expressed as an IC_50_ value.

### 3.3. Quantification of Apoptosis Using Propidium Iodide and Acridine Orange Double-Staining

TQ-induced cell death in CEMss leukemia cells was quantified using propidium iodide (PI) and acridine orange (AO) double-staining according to standard procedures and examined under a fluorescence microscope (Lieca attached with Q-Floro Software, Solms, Germany). Briefly, treatment was carried out in a 25-mL culture flask (Nunc, Roskilde, Denmark). CEMss cells were plated at a concentration of 2 × 105 cell/mL and treated with TQ at the IC_50_ concentration. Flasks were incubated in an atmosphere of 5% CO_2_ at 37 °C for 24 and 48 h. The cells were then spun down at 1,800 rpm for 10-min. Supernatant was discarded and the cells were washed twice using cold phosphate buffered saline (PBS) after being centrifuged at 1,800 rpm for 10-min to remove the remaining media. Five microliters of fluorescent dye containing AO (10 μg/mL) and PI (10 μg/mL) were added into the cellular pellet at equal volumes. Freshly stained cell suspension was dropped into a glass slide and covered by a cover slip. Slides were then observed under the fluorescence microscope within 30-min before the fluorescent colour started to fade. The percentages of viable, early apoptotic, late apoptosis and secondary necrotic cells were determined in >200 cells. AO and PI are intercalating nucleic acid-specific fluorochromes which emit green and orange fluorescence, respectively, when they are bound to DNA. Of the two, only AO is a membrane-permeable, cationic dye that binds to nucleic acids of viable cells and at low concentrations causes a green fluorescence. PI is impermeable to intact membranes but readily penetrates the membranes of nonviable cells and binds to DNA or RNA, resulting in orange fluorescence. When AO and PI are used simultaneously, viable cells fluoresce green and nonviable cells fluoresce orange under the fluorescence microscopy. The criteria for identification are as follows: (i) viable cells appear to have green nucleus with intact structure;(ii) early apoptosis exhibits a bright-green nucleus showing condensation of chromatin in the nucleus; (iii) dense orange areas of chromatin condensation showing late apoptosis; and (iv) orange intact nucleus depicting secondary necrosis [52].

### 3.4. Annexin V Assay

CEMss cells (2 × 105 cells/mL) were exposed to the IC_50_ concentration for 24 and 48 h and the Annexin V assay was performed using the BD Pharmingen TM Annexin V-FITC Apoptosis Detection Kit (APO Alert Annexin V, ClonTech). Briefly, treated cells were centrifuged for 10 min at 200 × g to remove the media. Later, the cells were rinsed with 1 × binding buffer supplied by the manufacturer. The rinsed cells were resuspended in binding buffer (200 μL) and annexin V (5 μL) and propidium iodide (10 μL) were added and the cells were then incubated at room temperature in the dark for 15 min. Flow cytometric analysis was carried out using a BD FACSCanto™ II instrument The binding buffer supplied by the manufacturer was used to bring the reaction volume to at least 500 μL for the flow cytometry analysis. DMSO-treated (0.1%, v/v) CEMss cells were used as control.

### 3.5. DNA Fragmentation Assay

Apoptosis was confirmed through detection of the fragmentation of chromosomal DNA with the DNA ladder method. The Apoptotic DNA Ladder Detection Kit (Chemicon International Inc., Palo Alto, CA, USA) was used to extract DNA from the cells. The cells were treated time dependently using IC_50_ concentration for 24, 12 and 6 h. Briefly, 2 × 105 cell/mL were washed twice using cool phosphate buffered saline (PBS) and the cells were collected by centrifuging them at 300 × g for 10-min. Following removal of the supernatant, the cells were lysed by adding 40 μL of TE (Tris and EDTA) lysis buffer, followed by 5 μL of Enzyme A (RNase A) and incubated at 37 °C for 10 min. Five microliters of Enzyme B (Proteinase K) were added and the lysate was further incubated at 50 °C for 30 min. Consequently, five microliters of ammonium acetate solution and 50 μL of isopropanol were added to the lysate, mixed well and kept at −20 °C for 10 min. The samples were then centrifuged for 10 min at 16,000 × g to precipitate the DNA. After washing, 70% ice cold ethanol was added to the DNA pellet. The pellet was then air dried and later dissolved in 30 μL of DNA suspension buffer. The extracted DNA samples were run on a 1.5% agarose gel in Tris-acetic acid-EDTA buffer. After electrophoresis, the gel was stained with ethidium bromide (Gibco BRL, Dakkopatts, Scotland) and the band obtained was visualized using a UV light transilluminator.

### 3.6. Caspase 3, 8 and 9 Activities

In vitro determination of the proteolytic activity of the enzymes in lysates of CEMss cells was performed. Induced apoptosis in cells by TQ at different times (12, 24 and 48 h) was detected using the R&D Systems Kit. Briefly, cells were collected, washed with cold PBS (1,800 rpm in 10 min) and subsequently resuspended in protein lysis buffer and incubated for 10 min on ice. Then, centrifugation was done at 10,000 g for 1 min, and the supernatant (protein) was collected. Fifty microliters of protein were transferred to 96-well plates in triplicates, then 50 μL of reaction buffer and 5 μL of caspase were added to each well and they were then incubated for 1–2 h at 37 °C. The plates were read using the microplate reader at a wavelength of 405 nm [45].

### 3.7. Cell Cycle Analysis

CEMss cells at a concentration of 2 × 105 cell/mL were cultured in RPMI 1640 medium containing 10% FBS and 1% penicillin/streptomycin seeded into a 25-mL culture flask (TPP Brand) and treated with TQ at IC_50_ concentration for 3, 6, 12, 24, 48 and 72 h. Following incubation, the cells were spun down at 1,800 rpm for 5 min. The supernatant was discarded and the pellet was washed with PBS (phosphate buffer saline) twice to remove any remaining media. To restore the integrity, a fixation of cell population for flow cytometry analysis was performed. Briefly, cell pellets were fixed by mixing 700 μL of 90% cold ethanol and keeping them at 4 °C overnight. The cells were then spun down at 200 g for 5 min and the ethanol was decanted. After being washed once with PBS, cells were resuspended in PBS (600 μL) and RNase (25 μL, 10 mg/mL) and propidium iodide (PI, 50 μL, 1 mg/mL) were added to the fixed cells and they were kept for 1 h at 37 °C. PI has the ability to bind to RNA molecules and hence, RNase enzyme was added in order to allow PI to bind directly to the DNA. The DNA content of the cells was then analyzed using the flow cytometer. The fluorescence intensity of sub-G1 cell fraction represented the apoptotic cell population.

### 3.8. Protein Detection by Western Blotting

#### 3.8.1. Extraction of Whole Protein from the Cells

CEMss cells at a concentration of 2 × 105 cells/mL were cultured in RPMI 1640 (PAA, Coelbe, Germany) medium containing 10% FBS, seeded into a 75-mm culture flask (TPP Brand) and then treated with IC_50_ concentration for 3, 6, 12 and 24 h. After incubation, the cells were spun down at 1,000 rpm for 10 min. The supernatant was discarded and the pellet was washed twice with phosphate buffered saline (PBS) to remove any remaining media. Estimation of the packed cell pellet volume was done and 20 volumes of mammalian cell lysis reagent (Proteo JET, Fermentas Life Sciences, Burlington, ON, Canada) were added to 1 volume of packed cells. The cells were then incubated for 10 min at room temperature on a shaker (900–1,200 rpm) and centrifugation was done at 16,000–20,000 × g for 15 min to clarify the lysate. The resultant lysate was then transferred to a new tube and stored at −70 °C until analysis by sodium dodecyl sulfate-polyacrylamide gel electrophoresis (SDS-PAGE).

#### 3.8.2. Western Blotting Analysis

Forty micrograms of protein extract was separated by 10% SDS-PAGE, transferred to a polyvinylidenedifluoride (PVDF) membrane (Bio-Rad, Hercules, CA, USA) and blocked with 5% non-fat milk in TBS-Tween buffer 7 (0.12 M Tris-base, 1.5 M NaCl, 0.1% Tween20) for 1 h at room temperature. Afterwards, it was incubated overnight with the appropriate antibody at 4 °C and then incubated with horseradish peroxidase-conjugated secondary antibody for 30 min at room temperature. The bound antibody was detected using peroxidase-conjugated anti-rabbit antibody (1:10000) or anti-mouse antibody (1:10000) followed by chemiluminescence and exposed by autoradiography. The primary antibodies β-actin (1:10,000), Bcl2 (1:1000), Bax (1:1000) and HSP70 (1:1000) were purchased from Santa Cruz Biotechnology Inc., Santa Cruz, CA, USA. 

### 3.9. Determination of Reactive Oxygen Species (ROS)

The production of intracellular ROS was measured using 2', 7'-dichlorofluorescin diacetate (DCFH-DA). Briefly, 10 mM DCFH-DA stock solution (in methanol) was diluted 500-fold in Hank's Balanced Salt Solution (HBSS) without serum or other additives to yield a 20 μM working solution. After 24 h of exposure to TQ, the cells in the 96-well black plate were washed twice with HBSS and then incubated in 100 μL working solution of DCFH-DA at 37 °C for 30 min. Fluorescence was then determined at 485-nm excitation and 520-nm emission using a fluorescence microplate reader (Tecan Infinite M 200 PRO, Männedorf, Switzerland) [43].

### 3.10. Statistical Analysis

Results were reported as means ± SEM of at least three analyses for each sample. Normality and homogeneity of variance assumptions were checked. Statistical analysis was performed according to the SPSS-16.0 package.

## 4. Conclusions

The anticancer activity of TQ in acute lymphoblastic leukaemia was studied by various methods including cell viability assay, AO/PI, DNA laddering, flow-cytometric analysis, caspase-3 activity and western blotting analysis. Results from all the experiments suggested that TQ could significantly inhibit proliferation and induce apoptosis in the leukemic cell line CEMss.
